# The multiple functions of the endocannabinoid system: a focus on the regulation of food intake

**DOI:** 10.1186/1758-5996-2-5

**Published:** 2010-01-21

**Authors:** Eduardo Tibiriça

**Affiliations:** 1 Laboratory of Cardiovascular Investigation, Oswaldo Cruz Institute, Fiocruz, Rio de Janeiro, Brazil

## Abstract

**Background:**

*Cannabis sativa *(also known as marijuana) has been cultivated by man for more than 5,000 years. However, there was a rise in its use in the 20^th ^century for recreational, religious or spiritual, and medicinal purposes. The main psychoactive constituent of cannabis, whose structure was identified in the 1960's, is Δ^9^-tetrahydrocannabinol. On the other hand, the discovery of cannabinoid receptors and their endogenous agonists took place only very recently. In fact, the first cannabinoid receptor (CB_1_) was cloned in 1990, followed 3 years later by the characterization of a second cannabinoid receptor (CB_2_). Since the 19^th ^century, the use of cannabis has been reported to stimulate appetite and increase the consumption of sweet and tasty food, sometimes resulting in significant weight gain. The recent description of the endocannabinoid system, not only in the central nervous system but also in peripheral tissues, points to its involvement in the regulation of appetite, food intake and energy metabolism. Consequently, the pharmacological modulation of the over-activity of this system could be useful in the treatment of the metabolic syndrome.

**Conclusions:**

The endocannabinoid system has important physiological functions not only in the central nervous system but also in peripheral tissues. The activation of central CB_1 _receptors, particularly in hypothalamic nuclei and in the limbic system, is involved in the regulation of feeding behavior, and especially in the control of the intake of palatable food. In the periphery, cannabinoid receptors are present in adipocytes, skeletal muscle, gastrointestinal tract and liver, modulating energy metabolism.

## Introduction

### Historical aspects

*Cannabis sativa *(marijuana or cannabis) has been cultivated by man since approximately 4,000 B.C [1,2]. At that time, the fibers obtained from the cannabis stems were mainly used to manufacture textiles and paper [1]. Moreover, from that time on, cannabis has also been known to have a variety of medicinal effects unrelated to its psychoactive properties, including effects on anorexia, emesis, pain, inflammation and neurodegenerative disorders [3]. Cannabis is the most widely used illicit drug in Western societies and also the one with the longest recorded history of human use. The popularity of marijuana as a recreational drug is due to its ability to alter sensory perception and cause elation and euphoria [2].

It has also been known since 300 B.C. that the recreational use of cannabis stimulates appetite, especially for sweet and palatable food [4,5]. Nevertheless, this phenomenon was seriously taken into consideration in biomedical research only in the last decade, after the description of the existence of an endogenous cannabinoid system [6,7], providing a physiological basis for the biological effects induced by cannabis and its derivatives.

Several chemical constituents of cannabis have already been identified, but its main psychoactive constituent is considered to be Δ^9^-tetrahydrocannabinol (Δ^9^-THC), whose structure was identified in the 1960's [8]. Even though several naturally-occurring agonists of the endogenous cannabinoid system have been known since then, the discovery of cannabinoid receptors and their endogenous agonists took place only very recently. In fact, the first cannabinoid receptor (CB_1_) was cloned in 1990 [9], followed 3 years later by the characterization of a second cannabinoid receptor (CB_2_) [10].

### The endocannabinoid system

Cannabinoid receptors belong to the G protein-coupled receptor superfamily and their activation modulates adenylate-cyclase, potassium and calcium channels and transcription factors such as mitogen-activated protein kinase [6,11]. The CB_1 _cannabinoid receptor is widely expressed in the central nervous system as well as in the periphery, while CB_2 _is mainly expressed in immune cells. In the central nervous system, CB_1 _is predominantly expressed presynaptically, modulating the release of neurotransmitters, including γ-aminobutyric acid (GABA), dopamine, noradrenaline, glutamate and serotonin [12].

**Figure 1:**
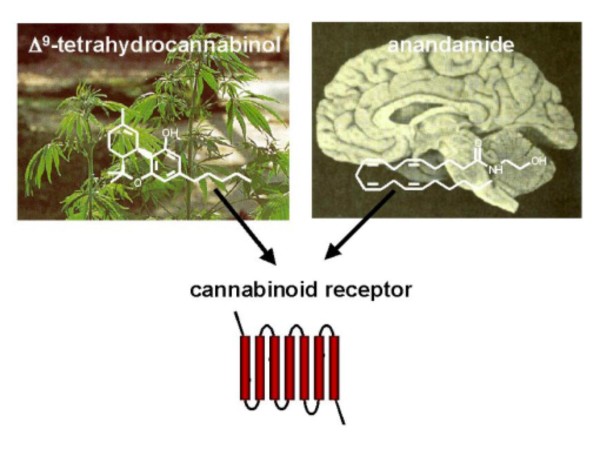
**Both Δ^9^-tetrahydrocannabinol, the psychoactive component of *Cannabis sativa*, and anandamide, an endogenous neurotransmitter in the human brain, bind to the same cannabinoid receptor**. (Photos/Diagrams from the Max Planck Institute of Psychiatry [42]).

The discovery of specific receptors mediating the actions of cannabis led to the search for endogenous ligands for cannabinoid receptors. The first endogenous cannabinoid, arachidonoyl ethanolamide, was identified in 1992 and was named anandamide, from the Sanskrit word 'ananda', meaning internal ecstasy [13,14]. Thus, both plant-derived (Δ^9^-THC) and endogenous (anandamide) agonists bind to the same cannabinoid receptors (Figure 1). Since the discovery of anandamide, other polyunsaturated fatty acid derivatives acting as functional agonists of cannabinoid receptors have been characterized and collectively termed endocannabinoids [15]. In contrast to classical neurotransmitters such as the catecholamines, endocannabinoids are not stored in the interior of synaptic vesicles because of the high lipophilicity of these ligands [6]. These findings led to the conclusion that the endocannabinoid system acts "on demand", meaning that the endocannabinoids are synthesized and released upon physiological or pathological stimulation [6].

### The endocannabinoid system and the regulation of food intake and energy metabolism

Since the 19^th ^century the use of cannabis has been reported to stimulate appetite and to increase the consumption of sweet and tasty food, sometimes resulting in significant weight gain [4,16]. The recent description of the endocannabinoid system, not only in the central nervous system but also in peripheral tissues, points to its involvement in the regulation of appetite, food intake and energy metabolism [17-20]. Numerous experimental data have confirmed this hypothesis and will be briefly summarized below, while clinical results will be presented in the next topic of this article.

### Endocannabinoid actions in the central nervous system

**Figure 2:**
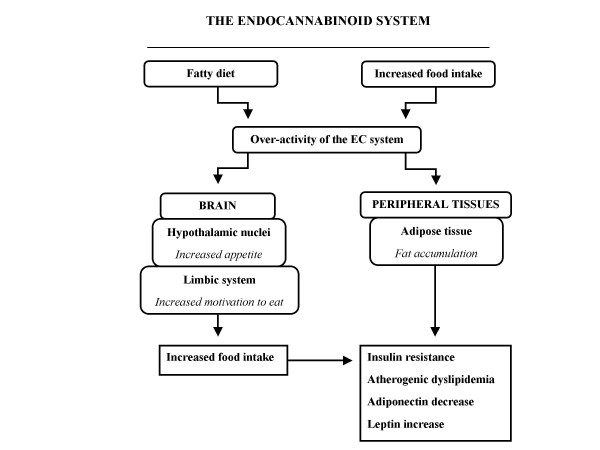
**The involvement of the endocannabinoid (EC) system over-activity in the pathophysiology of the metabolic syndrome**. Adapted from [20] and [17].

It has already been demonstrated that endocannabinoids initiate appetite by stimulation of CB_1 _receptors in hypothalamic areas involved in the control of food intake, such as the ventromedial hypothalamus (VMH) [21] (Figure 2). For instance, the injection of anandamide in the VMH of pre-satiated rats induces hyperphagia, which is prevented by previous hypothalamic administration of the selective CB_1 _cannabinoid antagonist rimonabant [22]. Using another experimental approach, Kirkham et al. [23] evaluated endocannabinoid levels in relation to the feeding behavior of rats in the hypothalamus and in the limbic forebrain, including the shell area of the nucleus accumbens, which is known to be linked to eating motivation. Endocannabinoid levels increased during fasting and declined during feeding, while no changes were detected in satiated rats. On the other hand, endocannabinoid levels in the cerebellum, which is not directly involved in the control of food intake, were unaffected by feeding behavior. Moreover, the injection of endocannabinoids into the nucleus accumbens induced feeding behavior, an effect that was also attenuated by rimonabant [23].

The experimental model of CB_1 _receptor knockout (CB_1_-/-) mice has also been used to clarify the involvement of the endocannabinoid system in the regulation of energy metabolism. An elegant study conducted by Ravinet-Trillou et al. compared CB_1_-/- knockout male mice with wild-type animals (CB_1+/+_) exposed either to a standard laboratory regimen or to a high-fat diet (HFD) [24]. When maintained on the standard diet, CB_1_-/- mice are lean and their body weight and adiposity are, respectively, 24 and 60% lower than that of CB_1+/+ _mice. CB_1_-/- mice submitted to the HFD do not develop obesity and do not display hyperphagia in contrast to CB_1+/+ _mice, and their feeding efficiency remains low. Furthermore, the insulin resistance that occurs in HFD-fed mice is not present in CB_1_-/- mice. These results led to the conclusion that CB_1 _receptors are involved not only in the development of diet-induced obesity but also in peripheral metabolic regulations [24]. These results were confirmed by Cota et al. [25], who showed that CB_1_-/- mice exhibit reduced spontaneous caloric intake and total fat mass, decreased body weight, and hypophagia, when compared with CB_1+/+ _littermates. Moreover, in young CB_1_-/- mice, the lean phenotype is predominantly caused by decreased caloric intake, whereas in adult CB_1_-/- mice, metabolic factors appear to contribute to the lean phenotype [25].

Another study carried out in mice submitted to HFD showed that a short-lasting reduction of food intake induced by a chronic oral treatment with rimonabant is dissociated from the long-lasting reduction of body weight and obesity. Moreover, rimonabant reverses the insulin resistance and lowered plasma leptin, insulin, and free fatty acid levels in this model of diet-induced obesity [26].

It is also noteworthy that Δ^9^-THC increases not only total food intake but also specifically increases the consumption of sweets, suggesting an interaction between the effect of the drug and the palatability of the food [27]. Another investigation confirmed this notion showing that different routes of Δ^9^-THC administration (oral, smoke inhalation or suppository) induced an effect on food selection [28]. Moreover, this selective action on food choice was confirmed in a number of animal studies, in which a potential role of cannabinoids in modulating the interaction of different pathways involved in the brain 'reward' system was hypothesized [29,30]. Thus, endocannabinoid signaling is involved orexigenically in both the homeostatic and the hedonistic control of food intake [31].

### Endocannabinoid actions in the periphery

#### Adipocytes

A functional CB_1 _receptor has already been identified in the fat tissue of rodents [25]. The CB_1 _receptor is expressed both in epididymal fat pads extracted from mice and in primary adipocyte cell cultures and seems to be involved in the regulation of lipogenesis. Moreover, the receptor is expressed in fat tissue obtained from CB_1+/+_, but not CB_1-/- _knockout, mice. CB_1 _agonists dose-dependently increase lipoprotein lipase activity in adipocyte cell cultures, and this effect can be blocked by the preincubation with the CB_1 _selective antagonist rimonabant, thus confirming a specific cannabinoid mechanism of action [25].

Interestingly, the blockade of CB_1 _receptors, either in vivo or in adipocyte cell cultures, results in a significant increase of adiponectin production [32]. Adiponectin is secreted exclusively by adipose tissue, with plasma levels negatively correlated with obesity [33]. Moreover, adiponectin is the only adipocytokine with anti-inflammatory effects and with a protective role against atherosclerosis, in addition to its insulin sensitizer effect [34]. The modulation of the endocannabinoid system using a chronic oral treatment with rimonabant simultaneously reduces body weight and stimulates adiponectin mRNA expression in adipose tissue of obese Zucker rats [32]. In parallel, the hyperinsulinemia associated with this experimental model was reduced by rimonabant treatment [32]. Moreover, adiponectin expression is up-regulated by the blockade of CB_1 _receptors only in CB_1+/+_, but not CB_1-/- _knockout, mice [32].

#### Skeletal muscle

It has already been shown that the modulation of the endocannabinoid system regulates glucose uptake through the phosphatidylinositol-3-kinase pathway in skeletal muscle cells [35]. Accordingly, the blockade of peripheral CB_1 _receptors could contribute to the improvement of glycemia observed in clinical trials with rimonabant [36]. In this context, Liu et al. clearly demonstrated that a sub-acute treatment with rimonabant increases soleus muscle glucose uptake in leptin-deficient obese mice [37].

#### Liver

The presence of CB_1 _receptors in the mouse liver has been confirmed using multiple methodological approaches [38], indicating that the hepatocyte could be a peripheral molecular target of the endocannabinoid system. In fact, the activation of the CB_1 _receptor increases the expression of lipogenic genes in the liver, which is the main source of de novo fatty acid synthesis in the body [38].

Synthetic agonists of the CB_1 _receptor induce the expression of several key lipogenic enzymes in liver, an effect that is prevented by a pretreatment with CB_1 _receptor antagonists [38]. Moreover, the basal rates of de novo fatty acid synthesis are markedly increased in CB_1+/+ _mice submitted to a high fat diet (HFD), compared with those of lean controls, but not in the CB_1-/- _knockout mice [38]. The pretreatment of CB_1+/+ _mice on HFD with a CB_1 _receptor antagonist reduces the rate of fatty acid synthesis [38]. In mice on HFD, the hepatic levels of the endocannabinoid anandamide were also greatly elevated. Finally, a marked deposition of lipid droplets was evident in wild-type, but not CB_1-/- _mice on HFD [38], suggesting the involvement of cannabinoid activation in liver steatosis.

## Conclusions

The endocannabinoid system is present and has important physiological functions not only in the central nervous system but also in peripheral tissues. The activation of central CB_1 _receptors, particularly in hypothalamic nuclei and in the limbic system, is involved in the regulation of feeding behavior, and especially in the control of the intake of palatable food. It is important to mention that the activation of the endocannabinoid system, in addition to increasing appetite (total food intake), also influences the palatability and the preferential choice of tasty and sweet foods [5] (Figure 2). Thus, endocannabinoid antagonists could be utilized for the treatment of the type of obesity associated with specific eating disorders such as 'sweet and snack-eating' and compulsive eating episodes.

In the periphery, cannabinoid receptors are present in adipocytes, skeletal muscle, gastrointestinal tract and liver, modulating energy metabolism (Table 1).

**Table 1:** Central and peripheral sites of action of CB_1 _receptor antagonists and outcomes of the blockade of the endocannabinoid (EC) system.

SITE OF ACTION	MECHANISMS OF ACTION	BLOCKADE OF EC SYSTEM EFFECTS
**Hypothalamic nuclei**	↓ food intake	body weight waist circumference

**Adipose tissue**	↑ adiponectin ↓ lipogenesis	dyslipidemia insulin resistance

**Skeletal muscle**	↑ glucose uptake	insulin resistance

**Liver**	↓ lipogenesis	dyslipidemia insulin resistance

**Gastrointestinal tract**	↑ satiety signals	body weight waist circumference

In conclusion, the endocannabinoid system seems to play a key role in the development and maintenance of obesity (see previous reviews [5,39,40]). In obese patients, excessive food intake, especially of sweet and palatable food, could lead to the hyperactivity of the endocannabinoid system, resulting in several metabolic alterations typical of the metabolic syndrome (Figure 1). Thus, the pharmacological modulation of the endocannabinoid system turns out to be a unique target in the treatment of those that are overweight or obese [41].

## Abbreviations

Δ^9^-THC: Δ^9^-tetrahydrocannabinol; CB_1_: type 1 cannabinoid receptor; CB_2_: type 2 cannabinoid receptor; VMH: ventromedial hypothalamus; HFD: high-fat diet.

## Competing interests

The author declares that they have no competing interests.

## Authors' contributions

ET conceived and wrote the manuscript. All authors read and approved the final manuscript.
